# QuEChERS Combined with Low-Temperature Partitioning and GC–MS as an Analytical Strategy for the Determination of Multiclass Pesticide Residues in Cocoa Beans

**DOI:** 10.1021/acsomega.5c10360

**Published:** 2025-12-17

**Authors:** Priscilla M. de Freitas Machado, Madson M. Nascimento, Paulo R. R. Mesquita, Manuela B. Nascimento, Lilian Lefol N. Guarieiro, Gisele O. da Rocha, Jailson B. de Andrade, Raildo M. de Jesus

**Affiliations:** † Departamento de Ciências Exatas, Universidade Estadual de Santa Cruz, Campus Soane Nazaré de Andrade, Rod. Jorge Amado, Km 16, Salobrinho, Ilhéus, Bahia 45662-900, Brazil; ‡ Secretaria da Agricultura, Pecuária, Irrigação, Pesca e Aquicultura, Centro Tecnológico Agropecuário do Estado da Bahia—CETAB, SEAGRI, Av. Milton Santos, 967—Ondina, Salvador, Bahia 40170-110, Brazil; § Universidade SENAI CIMATEC, Av. Orlando Gomes, 1845—Piatã, Salvador, Bahia 41650-010, Brazil; ∥ Universidade Federal da Bahia, Instituto de Química, Campus de Ondina, Salvador, Bahia 40170-115, Brazil

## Abstract

The QuEChERS (Quick, Easy, Cheap, Effective, Rugged, and Safe) technique has gained wide acceptance worldwide due to its efficiency in multiresidue pesticide analysis across diverse matrices. Continuous improvements have further enhanced its applicability and accuracy. In this study, QuEChERS was combined with low-temperature partitioning (LTP) for the simultaneous determination of 15 multiclass pesticide residues in cocoa beans by gas chromatography–mass spectrometry (GC–MS). A fractional factorial design (2^4–1^) was employed to optimize extraction parameters. The optimal conditions comprised 2.0 g of sample, 8.0 mL of acetonitrile and 2.0 mL of water as the extraction solvent, 150 mg of PSA sorbent, vortex extraction for 3 min at 2500 rpm, and partitioning for 24 h in a conventional freezer. The method was validated, with limits of detection and quantification ranging from 4.16 to 6.95 μg kg^–1^ and 13.9 to 23.1 μg kg^–1^, respectively. Recoveries were within 70–120%. The procedure was applied to 15 cocoa samples from Bahia and Pará, Brazil, and permethrin was the only pesticide detected, occurring in five samples. These results demonstrate that the proposed approach is effective and reliable, highlighting low-temperature partitioning as a simplified cleanup strategy for complex matrices such as cocoa.

## Graphical abstract

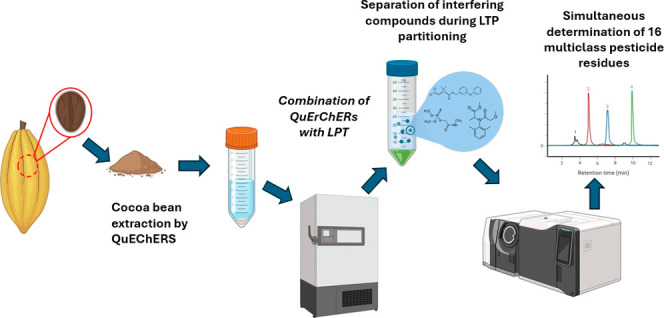

## Introduction

Cocoa (*Theobroma cacao* L.) is a tropical crop native to Central and South America, with a global production of approximately 5 million tonnes annually. Côte d’Ivoire and Ghana are the largest producers, while Brazil ranks sixth worldwide, led by the states of Bahia and Pará.^1,2^

Cocoa is best known as the primary raw material for chocolate but is also processed into products such as cocoa butter, biscuits, cocoa honey, and cocoa powder. Cocoa powder is derived from roasted, pressed, and milled nibs, containing on average 20% (w/w) lipids on a dry basis and less than 9% moisture.^3,4^

Cocoa cultivation faces significant phytosanitary challenges, including pests such as the red-banded thrips (*Selenothrips rubrocinctus*), brown rot (*Phytophthora* spp.), and the fungal pathogen *Moniliophthora perniciosa*, responsible for witches’ broom disease, which can reduce yields by up to 90% under favorable conditions.^5^ The spread of witches’ broom in the 1980s devastated cocoa production in southern Bahia, transforming the region from an exporter to an importer and causing profound socioeconomic impacts.^5^

Consequently, pesticides have become indispensable for pest management in cocoa plantations. These agrochemicals include organochlorines, organophosphates, carbamates, pyrethroids, and neonicotinoids, among others.^6,7^ However, their widespread use raises concerns due to potential adverse effects on human health and the environment. Pesticide exposure has been linked to ocular disorders, respiratory and endocrine disruptions, increased risks of cancer and neurodegenerative diseases, and reproductive toxicity.^8–12^

According to the Brazilian Health Regulatory Agency (ANVISA), pesticides such as atrazine, bifenthrin, cyproconazole, metalaxyl, tebuconazole, trifloxystrobin, and permethrin are approved for use in Brazil.^13^ However, among these, only metalaxyl, trifloxystrobin, and tebuconazole are authorized by the European Union.^14^ Thus, investigating these contaminants in cocoa samples is of utmost importance, as Brazil is one of the largest cocoa exporters, supplying cocoa commodities to South America, the United States, and the European Union.^2^ Furthermore, Organochlorine pesticides, in particular, belong to the class of persistent organic pollutants (POPs). Although banned in many countries, they continue to be used in some developing nations because of their chemical stability, resistance to degradation, volatility, and high lipophilicity, which promote bioaccumulation and neurotoxicity.^15^ Epidemiological evidence also associates their exposure with Parkinson’s disease, certain cancers, diabetes, and endometriosis.^16^

Residues of pesticides in cocoa have been reported in various studies.^17^ Analyzing pesticide residues in cocoa presents analytical challenges due to the matrix’s complexity, comprising high lipid content, fatty acids and esters, sugars, polyphenols, and caffeine. These components can interfere with extraction efficiency and contaminate analytical instrumentation.^18^ Therefore, efficient sample preparation methods incorporating effective cleanup steps are essential for accurate residue determination.

Several techniques have been employed for multiresidue pesticide analysis in cocoa. For instance, Idowu et al.^19^ quantified 14 organochlorines using Soxhlet extraction and silica/Na_2_SO_4_ cleanup, with GC ECD detection. Okoffo et al.^20^ determined 13 organophosphates and 9 pyrethroids employing SPE cartridges (Envi-carb/LC-NH_2_ and Bond Elute C18), acetonitrile extraction, and GC PFPD or GC ECD quantification. Yusiasih et al.^21^ applied dispersive SPE with PSA, Florisil, and MgSO_4_ for pyrethroids, followed by GC ECD and GC–MS.

Although these methods are effective, they are often laborious, costly, and generate substantial chemical waste. The QuEChERS (Quick, Easy, Cheap, Effective, Rugged, and Safe) method, first introduced by Anastassiades et al.,^22^ has become widely adopted for multiresidue analysis due to its simplicity and high recovery rates. QuEChERS typically involves acetonitrile extraction, followed by dispersive SPE cleanup with PSA and MgSO_4_. This approach has been successfully applied to matrices such as tomatoes,^23^ peppers,^24^ rice,^25^ soybeans,^26^ green vegetables,^27^ fruits,^28^ eggs,^29^ and cocoa.^30,31^ However, conventional QuEChERS protocols require multiple partitioning and cleanup steps, sometimes involving additional reagents.

Low-temperature partitioning (LTP) has emerged as an alternative, low-cost cleanup strategy. LTP entails adding a small volume of water (commonly ∼2 mL) to the extraction solvent—typically acetonitrile—and subjecting the mixture to freezing. During freezing, matrix interferences are retained in the aqueous phase while analytes remain in the organic phase.^32^ Depending on the matrix and analyte properties, mixtures with ethyl acetate or methanol can also be used.^33,34^ The combination of QuEChERS with LTP offers the potential to improve selectivity and reduce cleanup steps. While LTP has been applied to strawberries,^35^ tomatoes,^36^ lettuce,^37^ butter,^38^ and milk,^39^ to the best of our knowledge, no studies have yet evaluated the combination of QuEChERS and LTP for the determination of multiclass pesticides in cocoa matrices.

Therefore, this study aimed to evaluate the effectiveness of a QuEChERS–LTP analytical procedure for the determination of multiclass pesticide residues in cocoa beans. The factors that affect the QuEChERS–LTP procedure efficiency were optimized. After validation, the procedure was employed to investigate the occurrence of 15 multiclass pesticides in cocoa samples.

## Results and Discussion

### Optimization of QuERChERs–LTP Procedure

In this study, the factors evaluated were water volume, vortex agitation time, and both the type and mass of sorbent (Table S1). According to the Pareto chart of standardized effects (Figure 1A), only water volume had a positive and statistically significant effect on the responses. In contrast, extraction time, sorbent type, and sorbent mass were not significant at the 95% confidence level.

**Figure 1:**
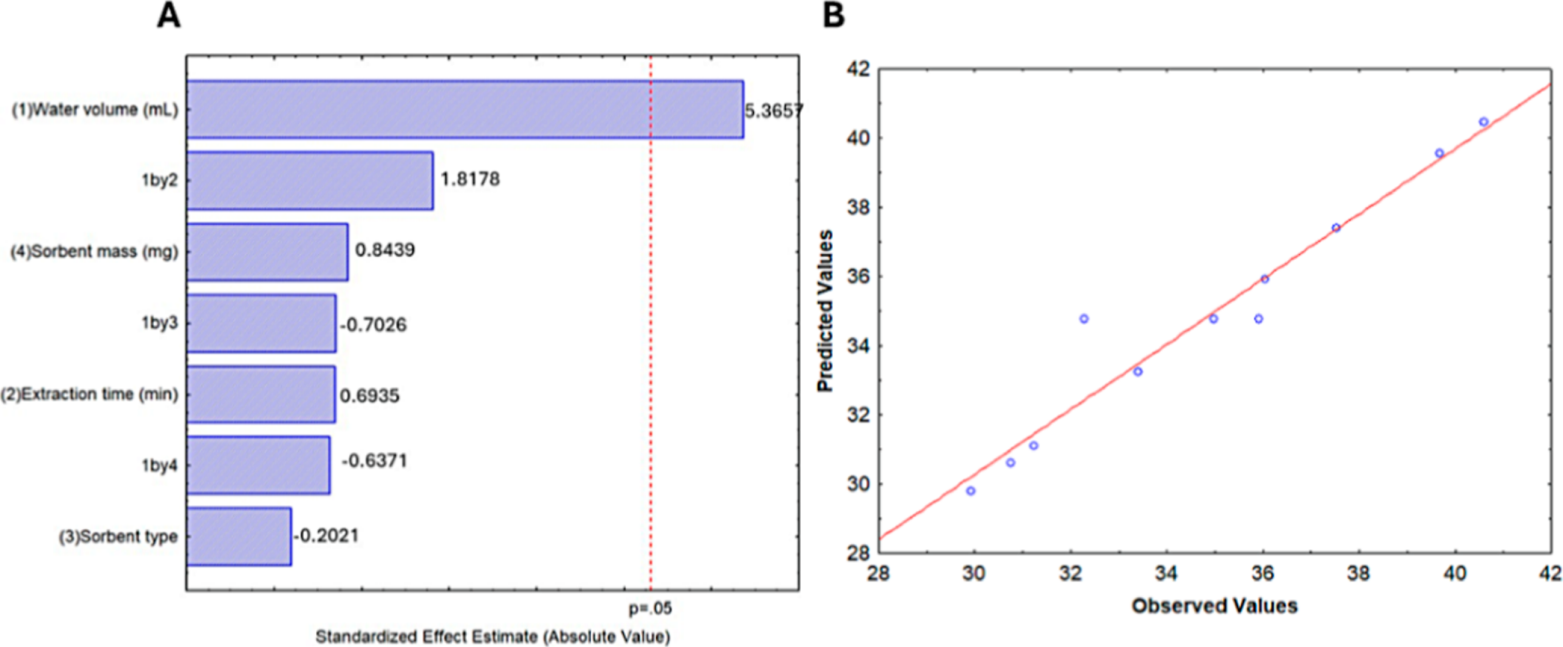
(A,B) Pareto chart of standardized effects obtained from the fractional factorial design of resolution IV (2^4–1^).

The fitted linear model showed no lack of fit at the 5% significance level, and the residuals were low and randomly distributed (Figure 1B). Although sorbent type and mass did not significantly affect the response, extracts obtained with PSA appeared clearer (Figure 2). Therefore, 150 mg of PSA and a vortex agitation time of 3 min were selected for subsequent experiments.

**Figure 2:**
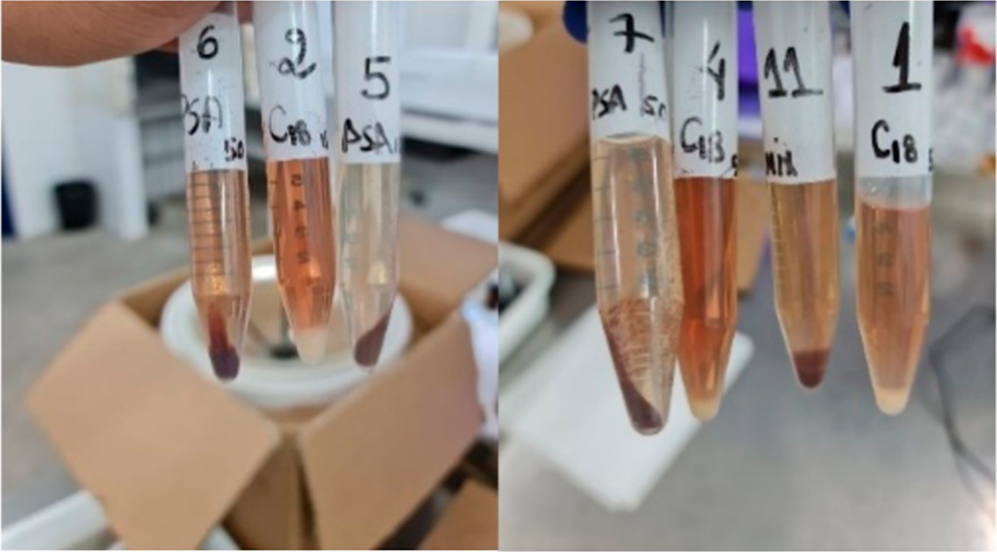
Extracts purified with PSA and C18 sorbents. Experiments: 1 (C18), 2 (C18), 4 (C18), 5 (PSA), 6 (PSA), 7 (PSA), 11 (C18/PSA).

The miscibility of water in acetonitrile, combined with their distinct freezing points (0 °C and −45 °C, respectively), enables the retention of undesirable polar components during freezing, thereby minimizing interference of coeluting compounds from cocoa matrix. Based on these considerations, the factor “water volume” was further studied in a univariate way at levels of 2.0 and 3.0 mL.

The results exhibited in Figure S1 show that recovery values for most pesticides remained within the acceptable range when 2 mL of water was added to the samples, whereas 3 mL led to overestimated recoveries, frequently exceeding 115%. This effect can be attributed to the strong affinity of water for phenolic compounds present in cocoa^4^ which, at high concentrations, may cause matrix-induced signal enhancement.^40^ Based on these findings, the optimal water volume was set at 2.0 mL.

### Evaluation of the Effect of Low and Ultralow Temperatures on the Analytical Response

Studies reporting the use of LTP generally employ a conventional freezer, which typically reaches −18 ± 2 °C as the minimum temperature. In this equipment, effective phase separation can require 8 to 24 h.^35–37^ In contrast, ultralow temperature freezers (ultrafreezers) can reach −80 °C, leading to a substantial reduction in partitioning time. In this study, tests performed with the conventional freezer achieved satisfactory partitioning after 24 h, whereas in the ultrafreezer, complete partitioning was observed in only 20 min.

Considering the analytical response in terms of peak area (Figure 3), higher signals were obtained when LTP was performed in a conventional freezer rather than an ultrafreezer. This behavior can be attributed to the partitioning mechanism.^32^ Partitioning depends on cooling and the subsequent freezing of the aqueous phase. In the conventional freezer, the slower freezing rate provides sufficient time for the system to approach thermodynamic equilibrium, favoring analyte diffusion between phases. Under these conditions, analyte molecules have more time to migrate and distribute optimally before complete solidification. In contrast, the rapid freezing in the ultrafreezer may trap analytes in a nonideal distribution, reducing the efficiency of the partitioning process^32^ (Figure 3). According to these observations, the conventional freezer was selected for subsequent experiments.

**Figure 3:**
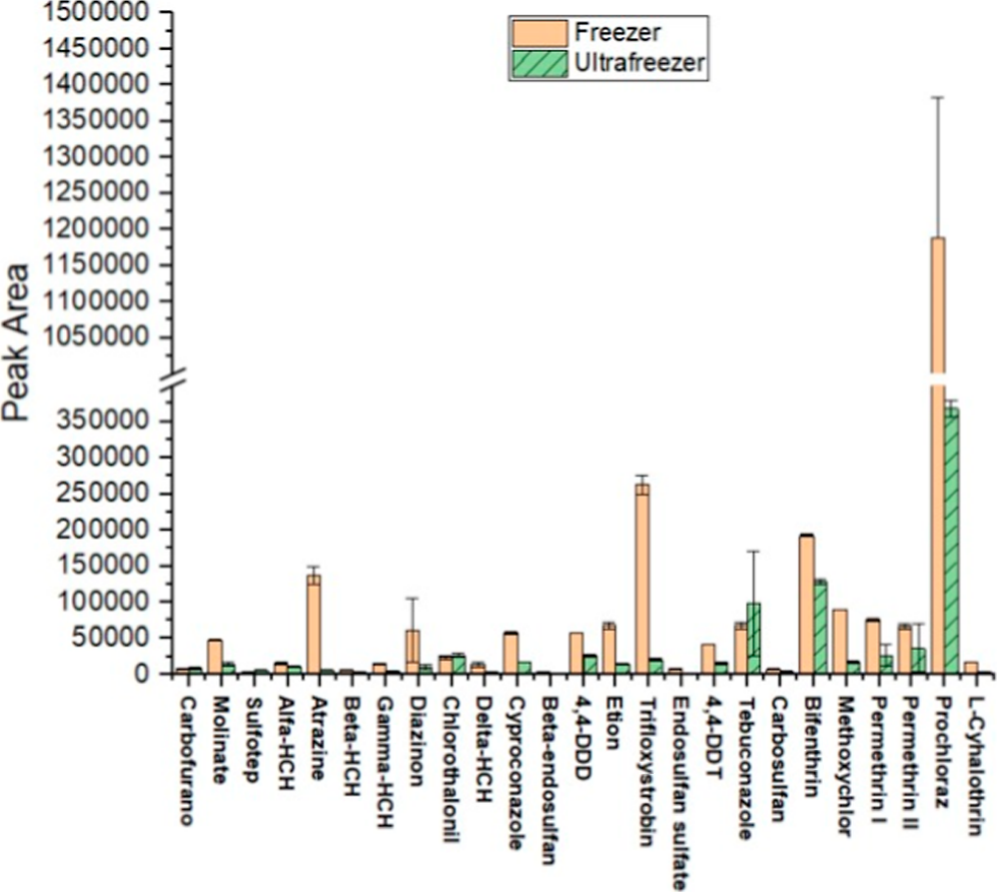
Comparison of chromatographic peak areas of pesticides after partitioning in the conventional freezer versus the ultrafreezer.

### Validation of the Analytical Procedure

The performance parameters achieved during validation of the analytical methodology are presented in Table 1.

**Table 1:** Validation Parameters of the Developed Analytical Procedure

pesticide	linear range (μg kg^–1^)	*R* ^2^	linearity (*p* < 0.05)	LOD (μg kg^–1^)	LOQ (μg kg^–1^)	MRL (μg kg^–1^)	repeatability RSD (%) (250 μg kg^–1^)	intermediate precision RSD (%) (250 μg kg^–1^)	ME
molinate	16.0–500	0.9993	-	4.78	15.9	50	5.7	13.4	6.45
α-HCH	17.6–500	0.9993	-	5.27	17.6	10	8.3	19.5	2.47
atrazine-*d*_5_ ^a^	24.4–500	0.9992	-	7.32	24.4	-	14.6	17.2	—
atrazine	18.1–500	0.9991	-	5.42	18.1	100	11.8	14.3	1.61
diazinon	23.1–500	0.9990	0.0520	6.95	23.1	50	8.6	14.0	9.01
disulfoton	19.2–500	0.9991	-	5.75	19.2	50	7.7	13.8	16.5
dimethachlor	21.8–500	0.9993	-	6.53	21.8	50	12.7	17.6	4.33
metalaxyl	31.2–500	0.9985	0.3704	4.77	15.9	50	8.8	20.8	17.6
*p*,*p*′-DDE	18.5–500	0.9992	-	5.55	18.5	50	5.7	13.1	3.17
cyproconazole I	22.4–500	0.9990	0.3337	6.70	22.4	50	9.6	17.0	20.5
cyproconazole II	22.0–500	0.9991	-	6.61	22.0	50	11.3	16.6	17.6
*p*,*p*′-DDD	14.9–500	0.9992	-	4.46	14.9	50	13.8	19.3	3.70
ethion	15.8–500	0.9991	-	4.73	15.8	50	15.7	19.9	10.6
trifloxystrobin	13.9–500	0.9995	-	4.16	13.9	50	10.9	18.6	9.64
tebuconazole	17.2–500	0.9991	-	7.30	17.2	50	8.4	18.1	18.3
bifenthrin	21.2–500	0.9990	0.0609	6.40	21.2	50	7.8	14.6	13.4
permethrin I	18.2–500	0.9991	-	5.50	18.2	100	10.4	11.8	2.30
permethrin II	16.6–500	0.9990	0.2221	4.97	16.6	100	7.7	14.6	19.6

a Surrogate standard.

The determination coefficients (*R* ^2^) obtained for all matrix-matched calibration curves were satisfactory, ranging from 0.9985 (metalaxyl) to 0.9995 (trifloxystrobin). The calibration curves for diazinon, metalaxyl, cyproconazole I, and permethrin exhibited *R* ^2^ values <0.9990 and were therefore evaluated by ANOVA (*p* < 0.05). The results indicated no evidence of lack of fit for these curves.

The obtained LOD and LOQ values ranged from 4.16 to 6.95 μg kg^–1^ and from 13.9 to 23.1 μg kg^–1^, respectively. Recent studies have reported concentrations of some target pesticides in cocoa samples within the range of 10.0–200 μg kg^–1^.^19,41^ This comparison indicates that the LOQ values achieved with the proposed method are adequate for the quantification of pesticide residues in cocoa products. Moreover, with the exception of α-HCH, the LOQs fall within the maximum residue limits (MRLs) established by European Union regulations.^42^

With respect to precision, the RSD values for repeatability and intermediate precision ranged from 5.7% to 15.7% and from 13.1% to 20.8%, respectively. Accordingly, all pesticides investigated in this study exhibited RSD values below 21% for both repeatability and intermediate precision, which is considered acceptable at the evaluated concentration levels.^43^

Regarding ME, it was observed that all studied pesticides exhibited ME > 1.10, which indicates a signal enhancement induced by the cocoa matrix. The values for ME ranged from 1.61 (atrazine) to 20.5 (cyproconazole) (Table 1). The ME arises primarily from competition between analytes and matrix constituents for active sites within the chromatographic system, particularly the silanol groups present in the injector.^44^ A commonly employed strategy to mitigate ME involves constructing calibration curves in the presence of analyte protectants.^45–47^ These protectants, compounds that simulate matrix components, interact with active sites in the chromatographic system, such as silanol groups, through hydrogen bonding.^45,48^ Consequently, the signal of the analyte in the presence of analyte protectants is significantly increased. However, in this study, calibration curves prepared in an analyte-free cocoa matrix solution provided a better analytical response than those prepared in an analyte protectant solution in acetonitrile (Figure 4). Therefore, matrix-matched calibration curves were selected to correct for the matrix effect instead of analyte protectants.

**Figure 4:**
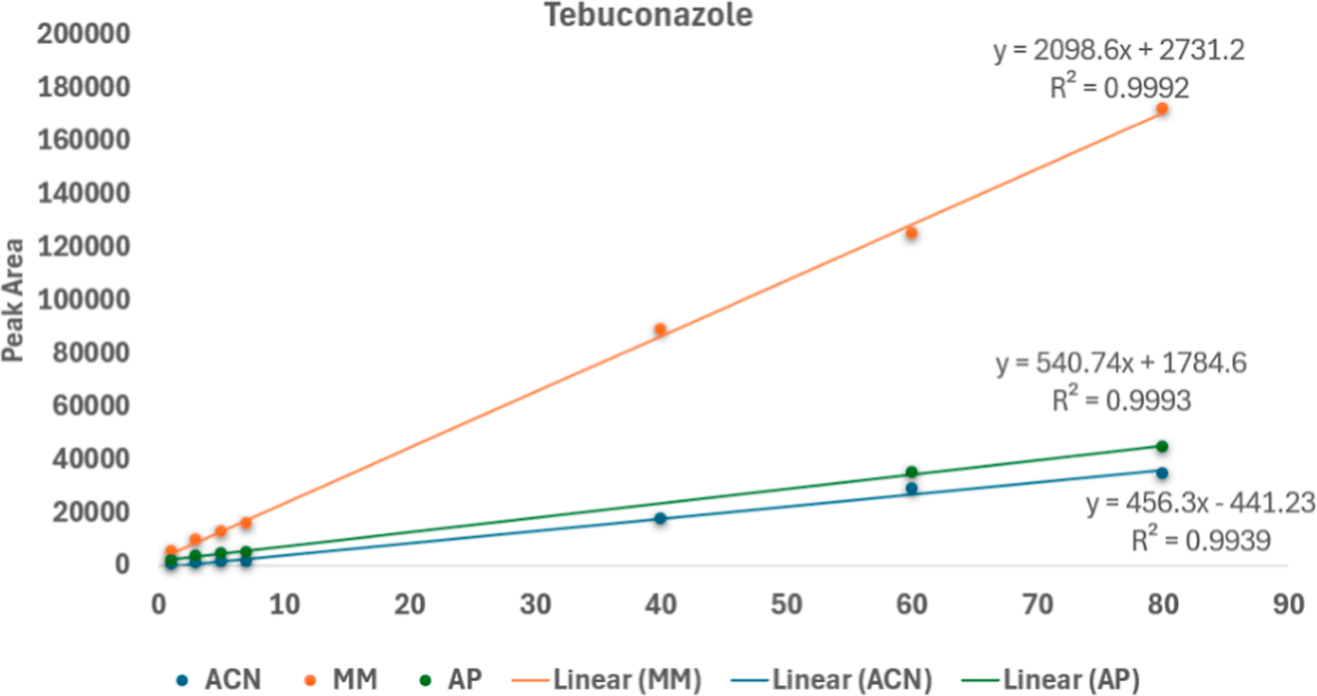
Influence of matrix components on the analytical response of tebuconazole calibration curves prepared in solvent, matrix extract, and with analyte protectants.

Concerning trueness, recoveries at the lowest concentration level (25 μg kg^–1^) ranged from 62.4% (dimetachlor) to 117% (metalaxyl), whereas at the highest concentration level (50 μg kg^–1^), they ranged from 81.8% (cyproconazole) to 120% (metalaxyl) (Table 2). Depending on matrix complexity and analyte concentration in the spiked sample, recoveries between 60% and 120% are generally considered acceptable. Therefore, for cocoa beans, this analytical procedure was considered accurate.

**Table 2:** Recovery (%) and Standard Deviation for Spiked Cocoa Samples (*n* = 3)

	added concentration	
pesticide	25 μg kg^–1^	50 μg kg^–1^
molinate	96.8 ± 0.9	102 ± 2
α-HCH	87.0 ± 7.8	98.1 ± 4.0
atrazine-*d*_5_	101 ± 4	109 ± 6
atrazine	87.3 ± 8.2	107 ± 1
diazinon	70.9 ± 0.5	88.5 ± 3.8
disulfoton	97.6 ± 2.3	99.6 ± 2.3
dimethachlor	62.4 ± 7.3	113 ± 7
metalaxyl	117 ± 6	120 ± 8
*p*,*p*′-DDE	96.4 ± 3.2	97.3 ± 5.1
cyproconazole I	68.8 ± 3.5	81.8 ± 4.0
cyproconazole II	110 ± 4	100 ± 5
*p*,*p*′-DDD	70.2 ± 5.1	95.7 ± 5.8
ethion	86.9 ± 3.2	103 ± 4
trifloxystrobin	100 ± 3	102 ± 3
tebuconazole	100 ± 6	95.5 ± 2.4
bifenthrin	87.5 ± 2.3	99.2 ± 1.5
permethrin I	109 ± 11	100 ± 1.5
permethrin II	89.9 ± 2.7	98.8 ± 2.1

Table S4 presents a comparison between the analytical performance of the proposed QuEChERS–LPT procedure and conventional methods for pesticide extraction from cocoa beans. The proposed procedure provided LOQ and recovery values comparable to those reported for established extraction techniques, while enabling the simultaneous determination of a wide range of chemical classes, including chloroacetamides, strobilurins, phenylamides, organochlorines, organophosphates, pyrethroids, thiocarbamates, triazines, and triazoles. Moreover, the incorporation of LPT as a cleanup step represents a clear advantage, as it simplifies multiple operations and reduces reagent consumption. Nonetheless, the long partitioning time (24 h) required in a conventional freezer, along with the relatively low recoveries for polar pesticides such as dimetachlor and diazinon, must be considered limitations of this procedure.

### Application to Real Samples

Fifteen samples were analyzed in triplicate, comprising two from the State of Pará and 12 from the State of Bahia, located in the North and Northeast regions of Brazil, respectively. Among the 15 pesticides investigated, only permethrin isomers were quantified in four samples (Table 3). The herbicide molinate was also detected in three samples; however, its concentration was below LOQ. Permethrin isomers concentrations ranged from 17.1 ± 0.3 to 49.6 ± 1.8 μg kg^–1^. These values are below the MRL established by European Union regulation, which is 100 μg kg^–1^.^42^ Although permethrin is not permitted in the European Union,^42^ it remains authorized for use as insecticide in Brazil, particularly in crops such as cotton, rice, coffee, citrus, cabbage, beans, tobacco, maize, soybean, tomato, wheat, and grape.^13^ However, no MRL values have been established for cocoa beans under Brazilian legislation.^13^ Table 3 shows the concentration of detected compounds in the cocoa samples.

**Table 3:** Mean Concentrations (μg kg^–1^ ± Standard Deviation) of Target Pesticides Detected in This Study^a^

samples	molinate	permethrin I	permethrin II
BA	nd	47.9 ± 0.2	<16.6^b^
BAA	nd	nd	nd
BE	nd	<18.2^b^	<16.6^b^
BEP	nd	nd	nd
CAVE	nd	36.3 ± 1.7	<16.6^b^
CAM	nd	nd	nd
CW	nd	nd	nd
ILH	nd	46.2 ± 1.8	<16.6^b^
JD	nd	<18.2^b^	<16.6^b^
FL	nd	nd	nd
PALM01	nd	<18.2^b^	17.1 ± 0.3
PALM02	nd	nd	nd
PIT	<15.9^b^	nd	nd
St ANT	<15.9^b^	nd	nd
VIT	<15.9^b^	nd	nd

a Only compounds detected in at least one sample are reported.

b Concentrations < LOQ.

To assess method performance for pesticides not detected in the cocoa samples, one of the analyzed samples was fortified with a mixed standard solution at concentrations of 50, 250, and 500 μg kg^–1^. The fortified analytes were subsequently detected in the chromatograms presented in Figures S2–S4, indicating good response of the proposed method.

## Conclusions

The QuEChERS–LTP procedure was successfully developed for the determination of 16 multiclass pesticide residues in cocoa beans. For the first time, ultralow temperature (−80 °C) was applied to accelerate the low-temperature partitioning process during pesticide extraction from cocoa beans. Nevertheless, conventional freezing at −12 °C proved to be more effective for the extraction of the target analytes.

Evaluation of the matrix effect demonstrated that matrix-matched analytical curves, constructed in an analyte-free cocoa bean extract, were more effective in compensating for matrix interferences than the use of analyte protectants.

The pyrethroid permethrin was quantified in four cocoa samples. However, the concentrations did not exceed the MRL established by European Union legislation.

Overall, the QuEChERS–LTP procedure proved to be a promising approach for pesticide residue determination, as it requires smaller volumes of solvents and reagents, involves fewer cleanup and extraction steps, and shows strong potential for application to other complex matrices.

## Experimental Section

### Reagent and Materials

Individual stock solutions of analytical standards were prepared in methanol: atrazine-*d*_5_ (850 mg L^–1^), bifenthrin (1870 mg L^–1^), cyproconazole (1020 mg L^–1^), diazinon (1880 mg L^–1^), dimethachlor (1477 mg L^–1^), disulfoton (2450 mg L^–1^), ethion (2204 mg L^–1^), molinate (2010 mg L^–1^), permethrin (1824 mg L^–1^), and tebuconazole (1070 mg L^–1^). A mixed standard solution (Mix A) at 10 mg L^–1^ was prepared by appropriate dilutions. Another mixed solution (Mix B) containing atrazine, metalaxyl, and trifloxystrobin was also prepared in methanol (10 mg L^–1^). All standards were purchased from AccuStandard (New Haven, USA) with ≥97% purity.

An additional reference mixture containing 16 organochlorine pesticides (EPA 46960-U, Sigma-Aldrich, USA) was dissolved in hexane/toluene (2000 mg L^–1^) and diluted to 100 mg L^–1^ in hexane.

Acetonitrile (99.8%), methanol (99.9%), and *n*-hexane (98.5%) were obtained from Merck (Darmstadt, Germany). Sodium chloride (99.8%) was purchased from Sigma-Aldrich, and anhydrous sodium sulfate (99%) from Merck. Ultrapure water was produced using an ultrapure water system (18.2 MΩ cm, <3 ppb TOC; Merck Millipore, Germany). PSA sorbent (primary-secondary amine, 70 Å) was obtained from Sigma-Aldrich, and C_18_ sorbent (55–105 μm) from Waters (USA).

An analyte-free matrix solution was obtained by successive extraction of 2 g of dried cocoa beans with acetonitrile, followed by cleanup using the proposed analytical procedure. The resulting extract was injected into the GC–MS system to verify the presence of target pesticides, and no coeluting compounds were detected. This analyte-free solution was subsequently employed to prepare matrix-matched calibration curves with eight concentration levels (1.00–100 μg L^–1^).

### Instrumental Apparatus

Pesticide separation and identification were performed on a Shimadzu GC-MS-QP2010SE system (Kyoto, Japan) equipped with an AOC-20i autosampler. Separation was achieved using an Agilent DB-5MS capillary column (30 m × 0.25 mm × 0.25 μm) with a stationary phase of 5% phenyl/95% dimethylpolysiloxane. The instrumental conditions were based on Nascimento et al.^49^ and they are summarized in Table 4.

**Table 4:** GC–MS/SIM Instrumental Conditions Employed to the Determination of the Target Pesticides

parameter	condition/description
carrier gas	helium (99.999%)
flow rate	1.15 mL min^–1^ (constant flow)
injection mode	splitless (0.80 min)
injection volume	1.0 μL
injector temperature	280 °C
oven temperature program	95 °C (1 min), ramp 20 °C min^–1^ to 180 °C (2 min hold), ramp 5 °C min^–1^ to 250 °C, then ramp 5 °C min^–1^ to 300 °C
total run time	30.25 min
ionization mode	electron ionization (EI), 70 eV
ion source temperature	280 °C
transfer line temperature	280 °C
acquisition mode	selected ion monitoring (SIM)
monitored ions	three ions per analyte (one quantifier and two qualifiers)

For quantification, the base peak was used, while two additional ions were monitored for confirmation (Table S2) in accordance with SANTE guidelines.^50^ A GC–MS/SIM chromatogram illustrating the separation of all target pesticides is presented in Figure 5.

**Figure 5:**
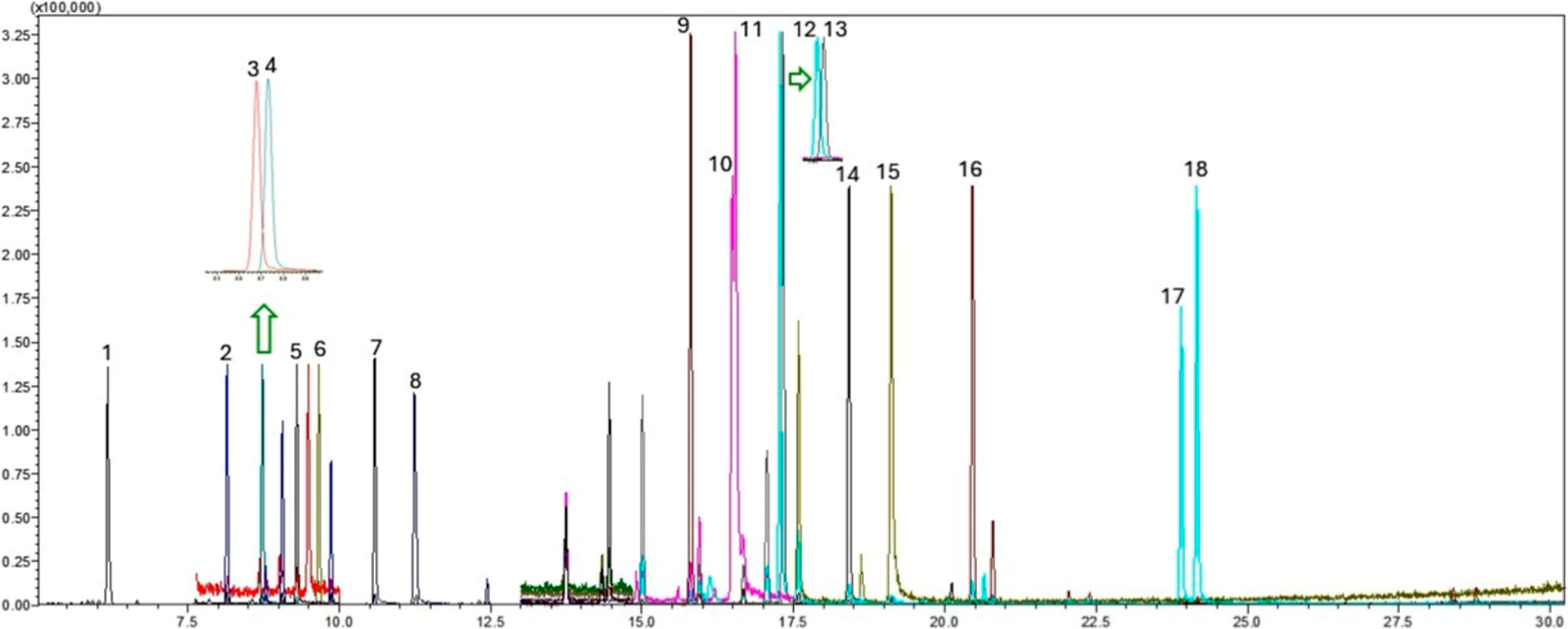
Extracted ion chromatogram (EIC) in SIM mode of the target pesticides obtained from the injection of a mixed standard solution at 100 μg L^–1^. Analytes are listed in order of elution: [1] Molinate (6.18 min); [2] α-HCH (8.15 min); [3] atrazine-*d*_5_ (8.69 min); [4] atrazine (8.75 min); [5] diazinon (9.31 min); [6] disulfoton (9.67 min); [7] dimethachlor (10.60 min); [8] metalaxyl (11.25 min); [9] *p*,*p*′-DDE (15.82 min); [10] cyproconazole I (16.50 min); [11] cyproconazole II (16.56 min); [12] *p*,*p*′-DDD (17.29 min); [13] ethion (17.34 min); [14] trifloxystrobin (18.43 min); [15] tebuconazole (19.13 min); [16] bifenthrin (20.47 min); [17] permethrin I (23.92 min); [18] permethrin II (24.18 min).

For compounds exhibiting stereoisomerism, such as cyproconazole and permethrin, multiple peaks corresponding to individual isomers were detected, each of which was integrated separately during data processing.^51^ The concentration reported in the samples represents the sum of all isomer contributions. The chemical structures of all pesticides analyzed in this study are presented in Table S3.

### Sampling and Sample Pretreatment

Fifteen cocoa samples were collected between 2023 and 2024 in Brazil, specifically from the municipalities of Ilhéus (14°47′20″ S, 39°02′58″ W) and Ituberá (13°43′58″ S, 39°08′15″ W) in Bahia, and from the state of Pará (1°27′0″ S, 48°30′0″ W). The cocoa beans were stored under refrigeration (−18 °C) until milling in an A11 benchtop grinder (IKA, São Paulo, Brazil). The ground material was then packaged in polypropylene bags and maintained under the same refrigeration conditions until analysis.

### Extraction Optimization and Clean-Up Procedure

The main factors influencing the extraction efficiency of pesticide residues in complex matrices such as cocoa include the volume and type of extraction solvent, ionic strength, sorbent mass and water volume, and extraction time.^20,32,52^

Following preliminary tests to assess the chemical system under study, a screening of variables was necessary to identify those with the significant effect on analytical response. Initially, a fractional factorial design of resolution IV (2^4–1^) was employed to evaluate these factors.^53^

The factors investigated to optimize the QuEChERS–LTP methodology combined with low-temperature partitioning for cocoa powder samples were water volume (0.5–2.0 mL), vortex extraction time (1.0–5.0 min), and sorbent mass (50.0–150 mg). To evaluate the effect of sorbent type on the cleanup efficiency of cocoa extracts, PSA and C18 were included in the experimental design matrix as categorical (qualitative) factors (Table S3). This approach has been adopted in recent studies.^54–56^ The total solvent volume was fixed at 10.0 mL, and the sample mass at 2.0 g. All experiments were performed in randomized order, including triplicates at the central point.

The peak area of each pesticide was used as experimental response. Due to the large number of analytes, a multiresponse approach was applied,^57^ as expressed in eq 1

1 $$RM=\frac{R\left(X1\right)}{LR\left(X1\right)}+\frac{R\left(X2\right)}{LR\left(X2\right)}+...+R\left(Xn\right)/LR\left(Xn\right)$$

where *R*(*Xn*) represents the peak area in a specific experiment and LR­(*Xn*) corresponds to the largest peak area observed among all experiments for that pesticide.

### Optimization of LPT in Freezer and Ultrafreezer

In the conventional freezing step, a Brastemp freezer (São Paulo, Brazil) operating at −12 ± 2 °C was used, and optimization was performed by varying the freezing time to 6, 12, and 24 h. For ultralow temperature partitioning, a laboratory ultrafreezer (Thermotemp Ultra Refrigeration, São Paulo, Brazil) set at −80 ± 2 °C was employed, with optimization conducted by testing freezing times of 10, 20, 60, and 120 min.

### Application of the Proposed Procedure to Real Samples

In a 50 mL Falcon tube, 2.0 g of sample was weighed using an analytical balance (AUX320, Shimadzu, Kyoto, Japan). A total of 2.0 g of salt (NaCl and Na_2_SO_4_ in a 1:1 w/w ratio) was added, followed by an aliquot of the atrazine-*d*_5_ solution (surrogate standard) to yield a final concentration of 250 μg kg^–1^. A mixture of acetonitrile (8.0 mL) and ultrapure water (2.0 mL) was then added. The system was vortexed (K40-10208, Kasvi, São Paulo, Brazil) for 3.0 min at 2500 rpm and centrifuged (MPW-351R, MPW Med. Instruments, Warsaw, Poland) for 10.0 min at 15 °C and 10,000 rpm. After phase separation, approximately 6.0 mL of the supernatant was transferred to a Falcon tube containing 150.0 mg of PSA sorbent. The mixture was vortexed again for 3.0 min at 2500 rpm and centrifuged for an additional 10.0 min under the same conditions. The tubes were then placed in the freezer for 24 h. Once the aqueous phase had frozen, the organic extract containing the analytes was collected, filtered through a Cytiva Whatman MiniUniprep G2 vial with a 0.20 μm membrane filter (Marlborough, Massachusetts, USA), and injected into the GC–MS system (Figure 6).

**Figure 6:**
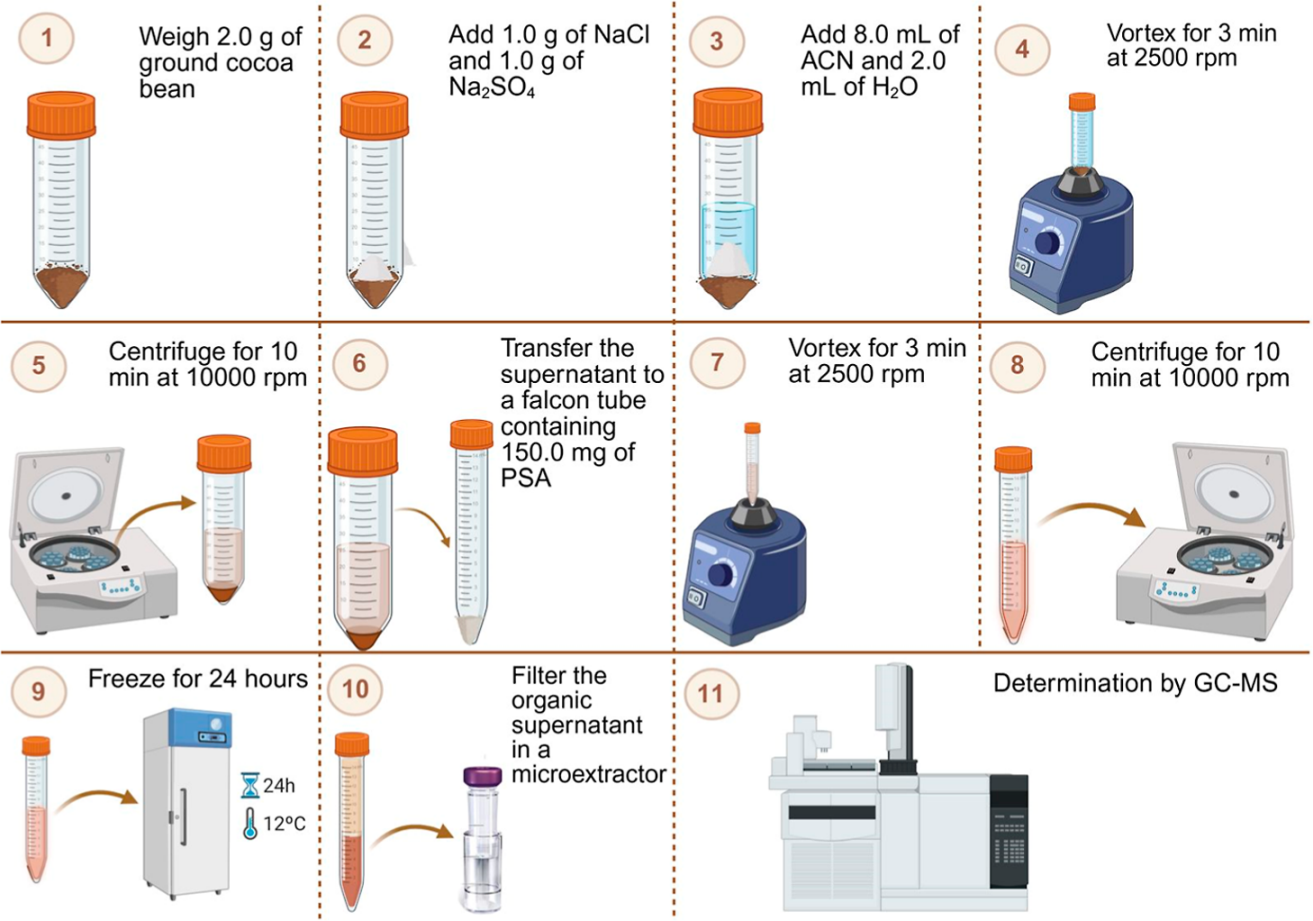
Schematic representation of the extraction procedure using the QuEChERS–LTP procedure for pesticide extraction in cocoa powder samples.

### Validation of the Analytical Procedure

The analytical method was validated following IUPAC guidelines for “In-house” validation.^58^ The following performance parameters were evaluated: selectivity, linear range, linearity, limit of detection (LOD), limit of quantification (LOQ), matrix effect (ME), precision and trueness.

Matrix-matched calibration curves were prepared in an analyte-free cocoa matrix solution at eight concentration levels, ranging from 1.00 to 100 μg L^–1^, with triplicate measurements at each level. To assess the matrix effect (ME), two additional calibration curves were constructed over the same concentration range: one in acetonitrile and another in a solution of analyte protectants (l-glucono-δ-lactone and sorbitol), 10 mg L^–1^ in methanol/ethyl acetate (1:1, v v^–1^).^48^

Linearity was evaluated according to significance of *R* ^2^, and the lack-of-fit test assessed by ANOVA (*p* < 0.05). The ANOVA was applied for curves with *R* ^2^ ≤ 0.9990.^43^

The limits of detection and quantification were calculated based on the calibration data, with LOD estimated as LOD = 3 × (SB/*a*) and LOQ as LOQ = 10 × (SB/*a*), where SB represents the standard deviation of the intercept and “*a*” is the slope of the calibration curve.^59^

The matrix effect (ME), reflecting the influence of coextracted components on the analytical signal, was evaluated by comparing the slopes of calibration curves prepared in solvent and in blank matrix extract. The ME was calculated as ME = slope_matrix_/slope_solvent_. An ME < 0.9 indicates signal suppression, while an ME > 1.1 indicates signal enhancement.^60^

Precision was expressed as repeatability and intermediate precision. Repeatability was assessed as the relative standard deviation (RSD) from ten consecutive injections of a single sample fortified with all analytes at 250 μg kg^–1^ (*n* = 10). Intermediate precision was determined by calculating the RSD of ten injections performed over three consecutive days (*n* = 30).

Owing to absence of certified reference materials for pesticides in cocoa beans, the trueness of the analytical procedure was assessed by spiking/recovery assays. For this purpose, 2 g of dried and milled cocoa samples were fortified at two concentration levels (25 and 50 μg kg^–1^) and processed according to the proposed method.

### Data Analysis

Experimental data were processed using Statistica 7.0 (Statsoft, USA) and OriginPro 2024 (OriginLab, USA). The obtained mathematical models for experimental design and linearity of the analytical curves were evaluated by analysis of variance (ANOVA, *p* < 0.05) at a significance level of 5% to assess both model significance and lack of fit.

## Supplementary Material



## Notes

Tables S1–S4 provide details on the fractional factorial design, the chromatographic and mass spectrometric parameters used for pesticide determination, the chemical information of the target pesticides, and a comparison of the analytical performance of the developed procedure. Figures S1–S3 present Box–Whisker plots representing the relative recoveries of the target pesticides, as well as chromatograms of spiked cocoa samples (PDF)
