# Supplementary material for: QuEChERS Combined with Low-Temperature Partitioning and GC–MS as an Analytical Strategy for the Determination of Multiclass Pesticide Residues in Cocoa Beans
Source: ACS Omega. 2025 Dec 17;10(51):63464–73. doi: 10.1021/acsomega.5c10360 (PMC12756727; doi:10.1021/acsomega.5c10360)
Supplement: Supplementary file 1 [file ao5c10360_si_001.pdf]

SUPPORTING INFORMATION

QuEChERS combined with low-temperature partitioning and GC–MS as an analytical strategy for the determination of multiclass pesticide residues in cocoa beans

Priscilla M. de Freitas Machado[a], *Madson M. Nascimento*[b,e], *Paulo R. R. Mesquita*[c], *Manuela B. Nascimento*[c], *Lilian Lefol N. Guarieirob*[e], *Gisele O. da Rocha*[d,e], *Jailson B. de Andrade*[b,e], *Raildo M. de Jesus*[a]*.

[a]Universidade Estadual de Santa Cruz, Campus Soane Nazaré de Andrade, Rod. Jorge Amado, Km 16 - Salobrinho, Ilhéus - BA, 45662-900.

[b]Universidade SENAI CIMATEC, Av. Orlando Gomes, 1845 - Piatã, 41650-010, Salvador – BA, Brazil.

[c]Secretaria da Agricultura, Pecuária, Irrigação, Pesca e Aquicultura – SEAGRI, Centro Tecnológico Agropecuário do Estado da Bahia - CETAB, Av. Milton Santos, 967 - Ondina, Salvador - BA, 40170-110.

[d]Universidade Federal da Bahia, Instituto de Química, Campus de Ondina, 40170-115 Salvador, BA, Brazil.

[e]Instituto Nacional de Ciência e Tecnologia em Energia e Ambiente - INCT, Universidade Federal da Bahia, 40170-115 Salvador, BA, Brazil.

SUPPLEMENTARY TABLES

Table S1. Fractional factorial design (resolution IV) applied to extraction optimization. The real values for each factor are shown between parentheses.

**Experiment**	**Factors**				**Response**
	Water volume (mL)	Extraction time (min)	Sorbent type	Sorbent mass (mg)	Multiple response
1	-1 (0.5)	-1 (1)	-1 (C_{18})	-1 (50)	30.759
2	1 (2.0)	-1 (1)	-1 (C_{18})	1 (150)	37.536
3	-1 (0.5)	1 (5)	-1 (C_{18})	1 (150)	31.233
4	1 (2.0)	1 (5)	-1 (C_{18})	-1 (50)	40.602
5	-1 (0.5)	-1 (1)	1 (PSA)	1 (150)	33.395
6	1 (2.0)	-1 (1)	1 (PSA)	-1 (50)	36.057
7	-1 (0.5)	1 (5)	1 (PSA)	-1 (50)	29.929
8	1 (2.0)	1 (5)	1 (PSA)	1 (150)	39.673
9	0 (1.25)	0 (3)	0 (C_{18}/PSA)	0 (100)	32.293
10	0 (1.25)	0 (3)	0 (C_{18}/PSA)	0 (100)	35.915
11	0 (1.25)	0 (3)	0 (C_{18}/PSA)	0 (100)	34.987

Table S2. Retention time (RT) and selected ions used for data acquisition in selected ion monitoring (SIM) mode.

**Agrotóxicos**	**T_R**	**Ion#1 Quantifier**	**Ion#2 Qualifier 1**	**Ion#3 Qualifier 2**
Molinate	6.18	126	55	187
Alfa-HCH	8.15	183	109	181
Atrazine-d_5	8.69	205	215	173
Atrazine	8.75	200	215	173
Diazinon	9.31	137	179	152
Disulfoton	9.67	88	60	274
Dimethachlor	10.6	134	197	148
Metalaxyl	11.25	160	206	146
p,p’-DDE	15.82	246	210	318
Cyproconazole I	16.50	222	222	235
Cyproconazole II	16.56	222	139	82
p,p’-DDD	17.29	235	165	199
Ethion	17.34	231	97	153
Trifloxystrobin	18.43	116	131	59
Tebuconazole	19.13	250	125	83
Bifenthrin	20.47	181	165	166
Permethrin I	23.92	183	165	163
Permethrin II	24.18	183	165	163

SUPPLEMENTARY TABLES

Table S3. Chemical structure, molar mass, chemical group, and classification of the studied pesticides.

**Pesticide**	**Molecular formula**	Structure	**MM g mol^{-1}**	**Chemical group**	**Classification**
p,p'-DDD	$C_{14}H_{10}Cl_4$	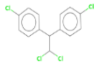	320.04	Organochlorine	Insecticide
p,p'-DDE	$C_{14}H_8Cl_4$	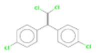	318.025	Organochlorine	Insecticide
Alpha-HCH	$C_6H_6Cl_6$	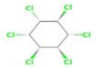	290.830	Organochlorine	Insecticide
Atrazine	$C_8H_{14}ClN_5$	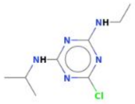	215.683	Triazine	Herbicide
Bifenthrin	$C_{23}H_{22}ClF_3O_2$	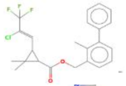	422.868	Pyrethroid	Insecticide, acaricide
Cyproconazole	$C_{15}H_{18}ClN_3O$	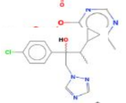	291.776	Triazole	Fungicide
Diazinon	$C_{12}H_{21}N_2O_3PS$	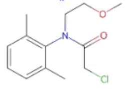	255.74	Organophosphorus	Insecticide, acaricide

Dimetachlor	$C_{13}H_{18}ClNO_2$		255.74	Chloroacetamide	Herbicide
Disulfoton	$C_8H_{19}O_2PS_3$	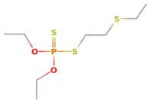	274.404	Organophosphorus	Insecticide, acaricide, fungicide
Ethion	$C_9H_{22}O_4P_2S_4$	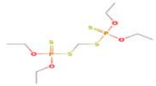	384.476	Organophosphorus	Inseticide
Metalaxyl	$C_{15}H_{21}NO_4$	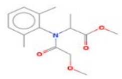	279.331	Phenylamide	Fungicide
Molinate	$C_9H_{17}NOS$	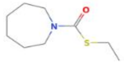	187.302	Thiocarbamate	Herbicide
Permethrin	$C_{21}H_{20}Cl_2O_3$	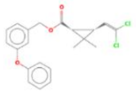	391.288	Pyrethroid	Insecticide, acaricide
Tebuconazole	$C_{16}H_{22}ClN_3O$	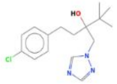	307.818	Triazole	Insecticide
Tryfluxostrobin	$C_{20}H_{19}F_3N_2O_4$	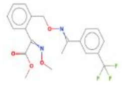	408.371	Strobirulin	Fungicide

Table S4. Comparison of the analytical performance of the developed procedure with other published studies using the cocoa matrix.

Ref.	Extraction technique	Chemical group	LOD (μg kg⁻¹)	LOQ (μg kg⁻¹)	Recovery (%)
This work	QuEChERS-LTP	Chloroacetamide, Strobilurin, Phenylamide, organochlorine, Organophosphate, Pyrethroid, Thiocarbamate, Triazine, Triazole	4.16 – 9.36	13.9 – 31.2	62.4 - 120
Zainudin et al. (2022)	d-SPE	Carbamate, Phenylamide, Isofthalonitrile, organochlorine, Organophosphate, Pyrethroid, Triazine, Triazole	NI*	10.0	83.2 - 119
Oyekunle et al. (2017)	Soxhlet	Organochlorine	0.017 – 0.405	NI*	80.1 - 109
Okoffo et al. (2017)	QuEChERS	Organophosphate and pyrethroid	10.0	NI*	70.0 - 100
Yusiasih et al. (2021)	d-SPE	Pyrethroid	NI*	10.0 – 30.0	86.0 - 117
Idowu et al. (2022)	Soxhlet	Organochlorine	NI*	6.0 - 107	95.2 - 100

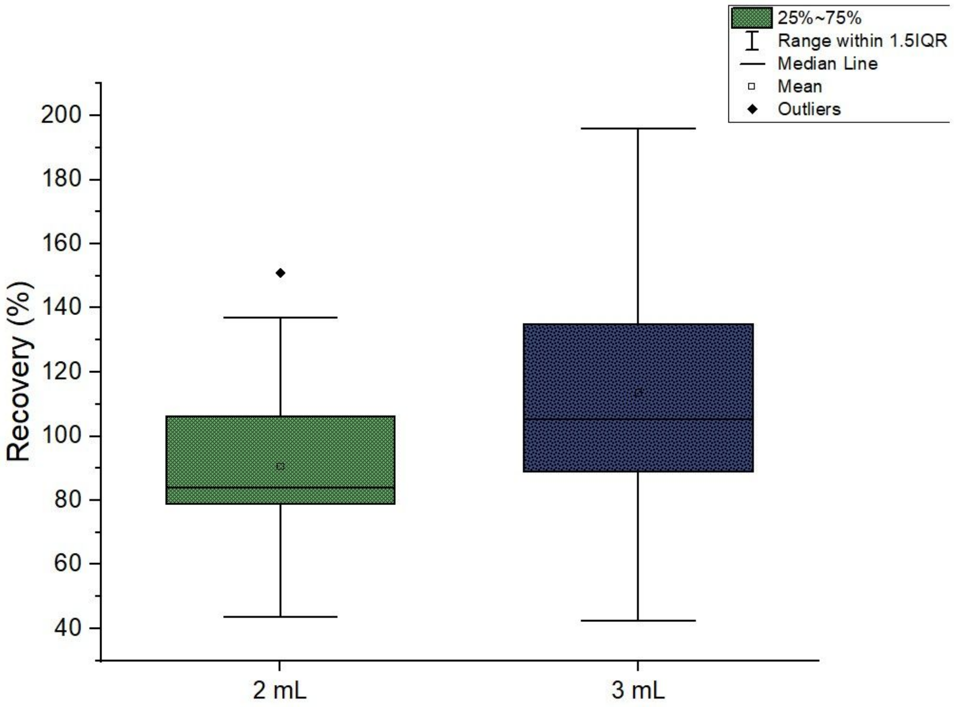

Figure S1. Box-Whisker plot representing the relative recoveries for all pesticides in different water volumes (2 and 3 mL).

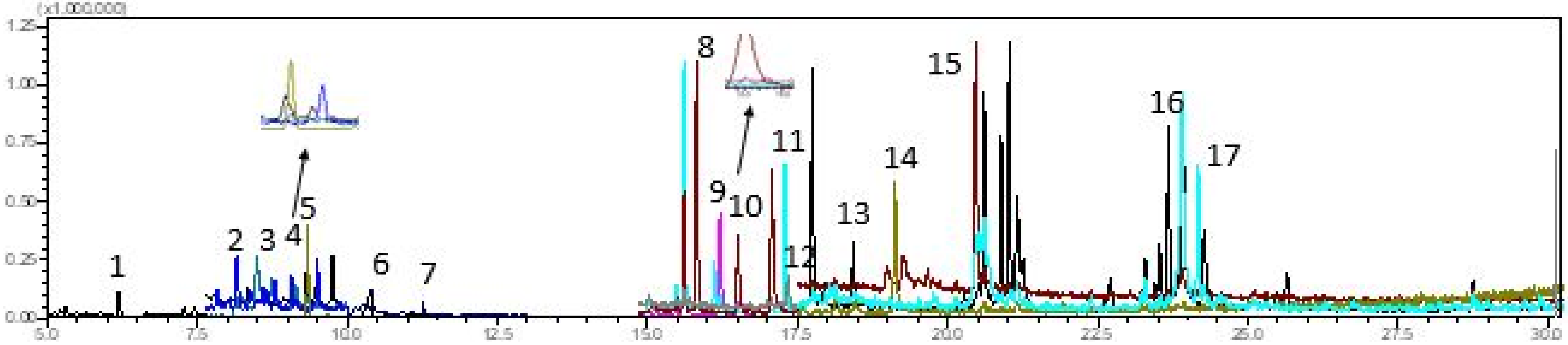

Figure S2. Chromatogram of the real sample (State of Bahia) fortified with the target pesticides at a concentration of 10 μg L^{-1}. Analytes are listed in order of elution: [1] Molinate (6.18 min); [2] α-HCH (8.15 min); [3] Atrazine (8.75 min); [4] Diazinon (9.31 min); [5] Disulfoton (9.67 min); [6] Dimethachlor (10.60 min); [7] Metalaxyl (11.25 min); [8] p,p′-DDE (15.82 min); [9] Cyproconazole I (16.50 min); [10] Cyproconazole II (16.56 min); [11] p,p′-DDD (17.29 min); [12] Ethion (17.34 min); [13] Trifloxystrobin (18.43 min); [14] Tebuconazole (19.13 min); [15] Bifenthrin (20.47 min); [16] Permethrin I (23.92 min); [17] Permethrin II (24.18 min).

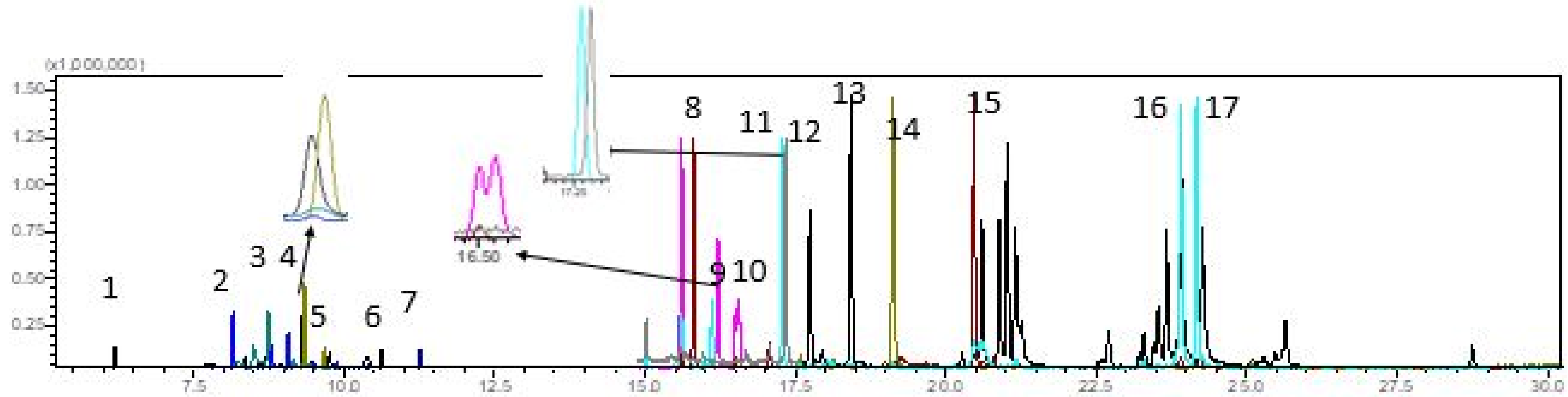

Figure S3. Chromatogram of the real sample (State of Bahia) fortified with the target pesticides at a concentration of 50 µg L^{-1}. Analytes are listed in order of elution: [1] Molinate (6.18 min); [2] α-HCH (8.15 min); [3] Atrazine (8.75 min); [4] Diazinon (9.31 min); [5] Disulfoton (9.67 min); [6] Dimethachlor (10.60 min); [7] Metalaxyl (11.25 min); [8] p,p′-DDE (15.82 min); [9] Cyproconazole I (16.50 min); [10] Cyproconazole II (16.56 min); [11] p,p′-DDD (17.29 min); [12] Ethion (17.34 min); [13] Trifloxystrobin (18.43 min); [14] Tebuconazole (19.13 min); [15] Bifenthrin (20.47 min); [16] Permethrin I (23.92 min); [17] Permethrin II (24.18 min).

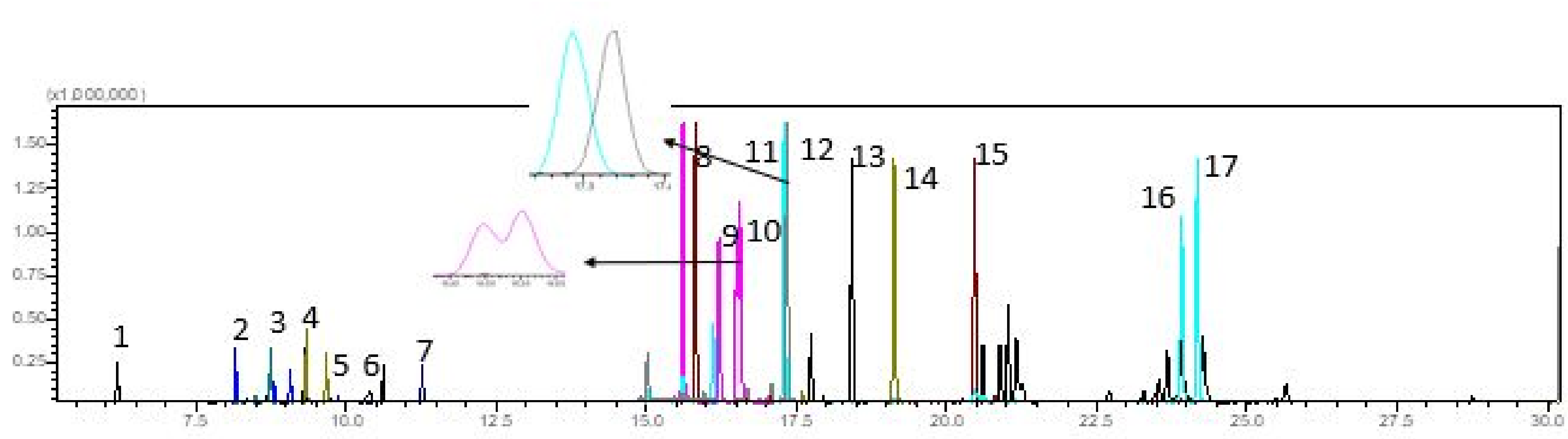

Figure S4. Chromatogram of the real sample (State of Bahia) fortified with the target pesticides at a concentration of 100 µg L^{-1}. Analytes are listed in order of elution: [1] Molinate (6.18 min); [2] α-HCH (8.15 min); [3] Atrazine (8.75 min); [4] Diazinon (9.31 min); [5] Disulfoton (9.67 min); [6] Dimethachlor (10.60 min); [7] Metalaxyl (11.25 min); [8] p,p′-DDE (15.82 min); [9] Cyproconazole I (16.50 min); [10] Cyproconazole II (16.56 min); [11] p,p′-DDD (17.29 min); [12] Ethion (17.34 min); [13] Trifloxystrobin (18.43 min);

REFERENCES

Idowu, G. A.; Aiyesanmi, A. F.; Oyegoke, F. O. Organochlorine Pesticide Residues in Pods and Beans of Cocoa (Theobroma Cacao L.) from Ondo State Central District, Nigeria. *Environmental Advances* **2022**, *7*. https://doi.org/10.1016/j.envadv.2021.100162.

Okoffo, E. D.; Fosu-Mensah, B. Y.; Gordon, C. Contamination Levels of Organophosphorus and Synthetic Pyrethroid Pesticides in Cocoa Beans from Ghana. *Food Control* **2017**, *73*, 1371–1378. https://doi.org/10.1016/j.foodcont.2016.11.004.

Yusiasih, R.; Pitoi, M. M.; Endah, E. S.; Ariyani, M.; Koesmawati, T. A. Pyrethroid Residues in Indonesian Cocoa Powder: Method Development, Analysis and Risk Assessment. *Food Control* **2021**, *119*. https://doi.org/10.1016/j.foodcont.2020.107466.

Oyekunle JAO, Akindolani OA, Sosan MB, Adekunle AS, Organochlorine pesticide residues in dried cocoa beans obtained from cocoa stores at Ondo and Ile-Ife, Southwestern Nigeria. Toxicol Rep, **2017**, 4:151–159. https://doi.org/10.1016/j.toxrep.2017.03.001

Zainudin, B. H.; Salleh, S.; Yaakob, A. S.; Mohamed, R. Comprehensive Strategy for Pesticide Residue Analysis in Cocoa Beans through Qualitative and Quantitative Approach. *Food Chem* **2022**, *368*. https://doi.org/10.1016/j.foodchem.2021.130778.
